# Regulatory Targets of the Response Regulator RR_1586 from Clostridioides difficile Identified Using a Bacterial One-Hybrid Screen

**DOI:** 10.1128/JB.00351-18

**Published:** 2018-11-06

**Authors:** Skyler D. Hebdon, Smita K. Menon, George B. Richter-Addo, Elizabeth A. Karr, Ann H. West

**Affiliations:** a Price Family Foundation Institute of Structural Biology, University of Oklahoma, Norman, Oklahoma, USA; b Department of Chemistry and Biochemistry, University of Oklahoma, Norman, Oklahoma, USA; c Department of Microbiology and Plant Biology, University of Oklahoma, Norman, Oklahoma, USA; Michigan State University

**Keywords:** bacterial one-hybrid assay, *Clostridioides difficile*, gene regulatory targets, response regulator, two-component signal transduction

## Abstract

The Clostridioides difficile R20291 genome encodes 57 response regulator proteins that, as part of two-component signaling pathways, regulate adaptation to environmental conditions. Genomic and transcriptomic studies in C. difficile have been limited, due to technical challenges, to the analysis of either high-throughput screens or high-priority targets, such as primary regulators of toxins or spore biology. We present the use of several technically accessible and generally applicable techniques to elucidate the putative regulatory targets of a response regulator, RR_1586, involved in sporulation of the hypervirulent C. difficile strain R20291. A DNA-binding specificity motif for RR_1586 was determined using a bacterial one-hybrid assay originally developed for Drosophila transcription factors. Comparative bioinformatics approaches identified and *in vitro* experiments confirmed RR_1586 binding sites upstream of putative target genes, including those that encode phosphate ion transporters, spermidine/putrescine biosynthesis and transport pathways, ABC type transport systems, known regulators of sporulation, and genes encoding spore structural proteins. Representative examples of these regulatory interactions have been tested and confirmed in Escherichia coli-based reporter assays. Finally, evidence of possible regulatory mechanisms is also presented. A working model includes self-regulation by RR_1586 and phosphorylation-dependent and -independent DNA binding at low- and high-fidelity binding sites, respectively. Broad application of this and similar approaches is anticipated to be an important catalyst for the study of gene regulation by two-component systems from pathogenic or technically challenging bacteria.

**IMPORTANCE** Clostridioides difficile spores survive under harsh conditions and can germinate into actively dividing cells capable of causing disease. An understanding of the regulatory networks controlling sporulation and germination in C. difficile could be exploited for therapeutic advantage. However, such studies are hindered by the challenges of working with an anaerobic pathogen recalcitrant to genetic manipulation. Although two-component response regulators can be identified from genetic sequences, identification of their downstream regulatory networks requires further development. This work integrates experimental and bioinformatic approaches, which provide practical advantages over traditional transcriptomic analyses, to identify the putative regulon of the C. difficile response regulator RR_1586 by first screening for protein-DNA interactions in E. coli and then predicting regulatory outputs in C. difficile.

## Abstract (precis)

Clostridioides difficile spores survive under harsh conditions and can germinate into actively dividing cells capable of causing disease. An understanding of the regulatory networks controlling sporulation and germination in C. difficile could be exploited for therapeutic advantage. However, such studies are hindered by the challenges of working with an anaerobic pathogen recalcitrant to genetic manipulation. Although two-component response regulators can be identified from genetic sequences, identification of their downstream regulatory networks requires further development. This work integrates experimental and bioinformatic approaches, which provide practical advantages over traditional transcriptomic analyses, to identify the putative regulon of the C. difficile response regulator RR_1586 by first screening for protein-DNA interactions in E. coli and then predicting regulatory outputs in C. difficile.

## INTRODUCTION

Two-component systems comprised of histidine kinases (HKs) and response regulators (RRs) are the primary means of signal transduction in bacteria. Stimuli, such as nutrient concentrations, antibiotic and environmental stresses, or quorum signals, stabilize active or inactive conformations of HKs through elaborate structural mechanisms (1). A kinase-active HK autophosphorylates a conserved histidine residue, which serves as a phosphoryl reservoir for the cognate RR. An RR is capable of phosphoryl transfer from the phosphohistidine residue of an HK and, to a lesser degree, from small-molecule phosphoryl donors to a conserved aspartate in the RR active site (2–4). Accommodation of the negatively charged phosphoryl group triggers conformational changes in the receiver domain, resulting in altered biological activity through an associated or downstream effector domain (5). RRs have been categorized into families by the identity or absence of an effector domain, the most abundant being the OmpR family (6), which regulates gene expression through a winged helix-turn-helix DNA-binding effector domain. Phosphorylation of OmpR family RRs often results in formation of a dimer with the DNA-binding domains (DBDs) that can recognize and bind direct repeats of specific DNA motifs oriented head to tail (7).

Bacterial genomes generally encode multiple RRs, each regulating a distinct biological process. The link between an RR and the process that it regulates can sometimes be inferred through homology or its genomic context. For example, the genome of the hypervirulent human pathogen Clostridioides difficile R20291 encodes homologues of VanR, KdpE, Spo0A, and EutV, which regulate vancomycin resistance, potassium starvation response, sporulation, and ethanolamine metabolism, respectively (8). These are important aspects of C. difficile biology and pathogenicity. C. difficile is an obligate anaerobe that can colonize the lower intestinal tract and causes symptoms ranging from diarrhea to fatal pseudomembranous colitis. Spores, formed after transduction of a signal through the master regulator Spo0A (9), are the primary means of transmission between hosts through the hostile aerobic environment. Inside the lower intestines, KdpE and EutV likely help C. difficile to compete with other microorganisms for efficient uptake and utilization of nutrients (10, 11). The roles of other important RRs in C. difficile have been identified experimentally (12–14). AgrA2 and CdtR are most notable because they regulate production of toxins A and B (13), the primary symptom-causing virulence factors, or C. difficile binary toxin (14), respectively. These, however, comprise only a fraction of the 57 total RRs in the C. difficile R20291 genome (8).

Several factors, including the intractability of modifying the C. difficile genome, have hampered the study of the remaining RRs. We therefore sought a means to accelerate the investigation of gene regulation by RRs in C. difficile R20291 using accessible, recombinant techniques. The regulatory effects of RRs on transcriptional machinery are mediated by the placement of RR-specific binding sites upstream of regulated genes. We employed a bacterial one-hybrid (B1H) assay and bioinformatics to characterize the DNA-binding specificity and predict the genomic binding sites of the previously uncharacterized OmpR family RR encoded by *CDR20291_1586* (RR_1586), which, along with other RRs, appears to be involved in processes important to sporulation (15). In the B1H assay (16), a chimera of the RNA polymerase ω subunit (ωRNAP) and a transcription factor bind to a randomized DNA sequence upstream of the *his3* and *ura3* genes. The weak *his3-ura3* promoter is not recognized by RNAP unless it is guided there by an interaction between the chimera (bait) protein and the upstream random DNA sequence (prey). The prey sequences from colonies surviving on histidine- and uracil-free medium contain the binding motif of the transcription factor, in this case, RR_1586. The putative regulon of RR_1586 was identified by searching for genes in the C. difficile R20291 genome with evolutionarily conserved binding sites. These findings are supported by an Escherichia coli-based green fluorescent protein (GFP) fusion reporter assay. We also report on the *in vitro* characterization of the effects of phosphorylation on oligomerization and DNA binding and propose a working model for gene regulation by RR_1586. We anticipate that similar analyses of other RRs and transcription factors could lead to a global understanding of gene regulation by RRs in C. difficile and other pathogenic bacteria.

## RESULTS

### RR_1586-DNA interaction specificity.

The DNA motif recognized by RR_1586 was identified using an E. coli-based bacterial one-hybrid (B1H) assay (16). In this assay, transcription of the *his3* and *ura3* genes is made possible if the bait protein binds to a 28-bp fragment inserted immediately upstream of the promoter. Binding of the bait chimera protein to both the prey DNA and the RNA polymerase enzyme (through the ωRNAP subunit) induces gene expression and cell survival in the absence of histidine and uracil. By plating millions of cells harboring a diverse library of randomized prey sequences on selective medium, a subset of prey sequences compatible with the bait chimera will survive. In a successful selection, this subset of sequences contains an overrepresentation of the transcription factor binding motif.

Although ωRNAP fusions of full-length RR_1586 and three DBD constructs of RR_1586 (Arg124, Ser131, Gln151) were tested, only the fusion at Ser131 yielded significant levels of selection. This position includes the predicted β platform (except for the first strand) and a winged helix-turn-helix of RR_1586. It does not include any of the linker leading to the receiver domain (see Fig. S1 in the supplemental material). The number of colonies that survived selection was only ∼7-fold higher than the background compared to that for the positive control, which achieved numbers at least 100-fold higher than the background (data not shown). This is probably because the zinc finger positive control has a much higher affinity to DNA than RRs belonging to the OmpR family and could indicate that the interaction affinity approaches the lower limit of detection for this assay.

**FIG 1:**
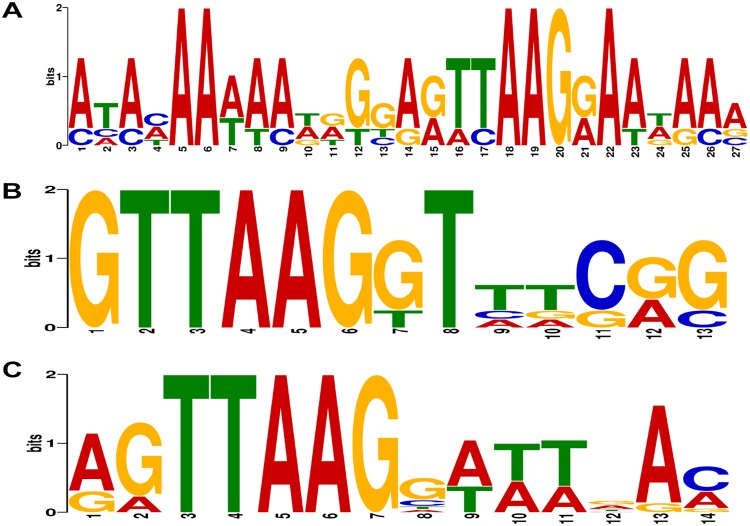
DNA-binding specificity of RR_1586. The motifs were overrepresented in colonies isolated from low-stringency (10 mM 3-AT) (A) or high-stringency (20 mM 3-AT) (B) selection or both data sets (C). Statistical confidence in these motifs increases with stringency and sample sizes. Associated E values are 3.1 × 10^−4^, 2.6 × 10^−15^, and 7.7 × 10^−24^, respectively.

Overrepresented motifs were identified in the randomized fragments from colonies that survived selection. More details of data processing, including the sequences analyzed, are available in the supplemental material (Fig. S2 and Table S1). Low-stringency selection with 10 mM 3-amino-1,2,4-triazole (3-AT) failed to produce a concise motif, whereas analysis of sequences from high-stringency screening or combined data sets produced highly significant motifs. The statistical significance of these motifs increased with the stringency of selection, as expected (Fig. 1) (16).

**FIG 2:**
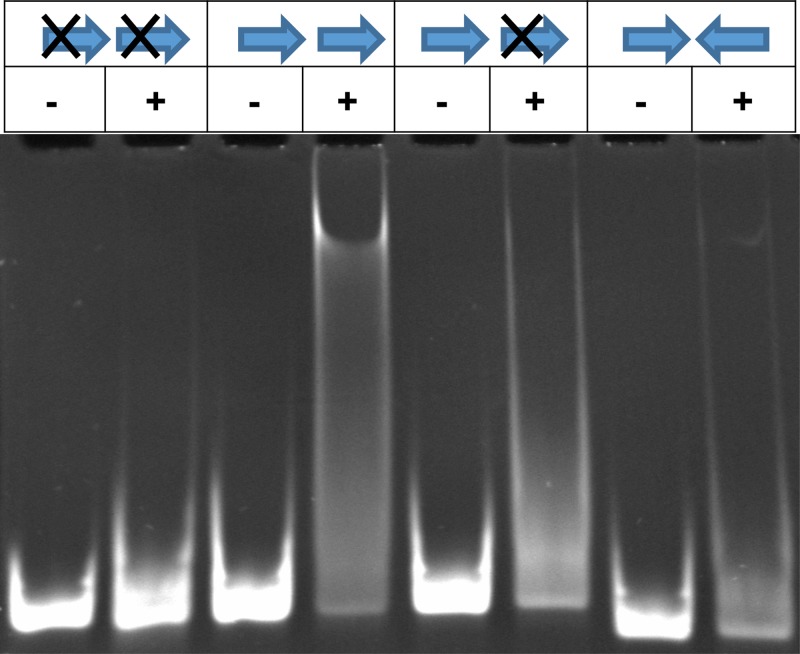
RR_1586 binds direct repeats *in vitro*. The presence of RR_1586 shifted direct repeats of the B1H-derived motif (arrow) in EMSAs. Inverting or substituting (cross) one or both of the repeats diminished the shift, suggesting a weakened interaction. The oligonucleotide sequences are listed in Table S2 in the supplemental material. The presence (+) or absence (−) of RR_1586 protein is indicated above each lane.

Binding of full-length RR_1586 to the observed motif was confirmed by electrophoretic mobility shift assays (EMSAs), as shown in Fig. 2. Given that OmpR family response regulators typically bind direct repeats and the observation that RR_1586 purifies as a dimer (see below), we presumed that the biological motif recognized by RR_1586 is a direct repeat of the B1H-derived motif. This was affirmed by EMSAs, where we observed a gel-shifted band pattern in the presence of RR_1586 for a direct repeat of the motif and, to a much lesser extent, for a single motif. A substituted repeat or an inverted repeat showed little to no gel shift in the presence of RR_1586, suggesting a weakened or no interaction. Here, we refer to a direct repeat of the first 11 nucleotides of the B1H-derived motif (Fig. 1C) as the RR_1586 binding site.

### Putative regulon of RR_1586.

Having characterized the DNA-RR_1586 interaction specificity, we used bioinformatics to identify a putative regulon and the biological functions associated with the RR_1586 binding site. The assumption of an evolutionarily conserved biological role of an RR suggests that the RR gene, its downstream target genes, and the associated *cis*-regulatory elements will be conserved. We used scripts from the Regulatory Sequence Analysis Tools suite (RSAT) (17) to evaluate the coconservation of these three elements relative to RR_1586 in the genomes of 26 species in the Peptostreptococcaceae family, to which C. difficile belongs. Orthologues of RR_1586, defined as bidirectional best BLASTP hits (18), were found in 17 genomes, which were further analyzed for the conservation of RR_1586 binding sites (Table S3). Fourteen putative gene targets (Table 1) were identified. These genes represent the operons that comprise the conserved core of the putative RR_1586 regulon, including seven operons that encode ion or ABC-type transporters.

**TABLE 1:** Locus tags of operon leaders and the upstream RR_1586 binding site

Operon leader	Annotation	Predicted RR_1586 binding site^*a*^	Justification
*CDR20291_2142*	Hypothetical protein	AA**TTAAG**GTATAA**TTAAG**TTTT	RSAT/exact
*CDR20291_3145*	Protease	AG**TTAAG**GTTTAA**TTAAG**ATTA	RSAT/exact
*CDR20291_0818*	SpeADEB	TT**TTGAG**TTTTAG**TAAGC**TTTT	RSAT
*CDR20291_0879*	PotABCD	AG**TAAAC**AAAATG**TTTAG**TAAA	RSAT
*CDR20291_1470*	Transcriptional regulator	AA**TCGAG**GGAAAG**TTAAC**AAAA	RSAT
*CDR20291_1527*	Hypothetical protein	AG**TTAAG**GTATAA**TTATT**TTAT	RSAT
*CDR20291_1565*	Hypothetical protein	AT**TTAAG**CTTTAT**TTAAG**GTTA	RSAT
*CDR20291_1626*	Na^+^/phosphate cotransporter	TA**TTAAT**GTTTTG**TTAAG**TATA	RSAT
*CDR20291_1855*	Tyrosine recombinase	AT**TTAGG**GAATAG**TTAGT**GATA	RSAT
*CDR20291_2009*	Na^+^/H^+^ antiporter	GG**ATATA**GAATAG**ATAAG**AAAA	RSAT
*CDR20291_2188*	Two-component system	TC**TTAAG**AAATAT**TTAAG**AATT	RSAT
*CDR20291_2890*	ABC transporter	AT**GTAAT**ATTTAC**TTAAG**GATT	RSAT
*CDR20291_3121*	Phosphate transport (pst)	TA**TTAGG**ATTAAG**TTAAG**CAAG	RSAT
*CDR20291_3239*	ABC transporter	TG**TAAAG**GATATA**TTAAG**ACAA	RSAT
*CDR20291_2468*	Neutral Zn metallopeptidase	AG**TTAAG**TGAATA**TTAAG**AGGA	Exact
*CDR20291_0571*	Peptidase	GA**TTAAG**TATGAA**TTAAG**CATG	Exact
*CDR20291_0578*	Chloride ion channel protein	TA**TTAAG**AATGGG**TTAAG**AGTA	Exact
*CDR20291_0610*	ATP-dependent peptidase	GA**TTAAG**TATTTA**TTAAG**TATT	Exact
*CDR20291_0884*	Signaling protein	TA**TTAAG**TATTTA**TTAAG**TAAA	Exact
*CDR20291_2143*	Signaling protein	AA**TTAAG**GTATAA**TTAAG**TTTT	Exact
*CDR20291_0477*	SleB	AA**ATAAG**CTAAAA**ATAAG**TAGA	Germination
*CDR20291_0523*	CotJC1	TA**TTAAA**TATATA**TTAAG**GAGG	Sporulation
*CDR20291_1583*	Hypothetical protein	AA**TTAAG**GAGCAA**TTAAA**TGAT	Autoregulation
*CDR20291_3401*	SpoIIR	TA**TTATG**AATAAA**TTAAA**TTTA	Sporulation
Consensus		DR**TTAAG**NWWWDR**TTAAG**NWWW	

a Boldface nucleotides represent the predicted binding sites.

Single-genome scanning revealed additional, nonconserved putative gene targets. The matrix-scan script in RSAT identified several hundred potential target operons with statistically significant matches (*P* < 0.0005) to the RR_1586 binding site. Negative controls using permuted motifs (matrix-quality script) suggested a high false-positive rate, probably due to the low GC content in both the genome and the RR_1586 binding site. We therefore manually selected several potential binding sites for further testing: ideal binding sites and sites upstream of sporulation/germination-associated genes or upstream of the *CDR20291_1583* operon (which includes the *CDR20291_1586* gene according to the DOOR2 operon database) (19). These 10 genes, along with the 14 genes mentioned above, are listed in Table 1.

The above-described bioinformatic analysis identified associations between the RR_1586 binding site and downstream genes. Further analysis using Gene Ontology for Motifs (GOMo) (20) identified significant associations between the presence of the RR_1586 binding site and the biological functions of downstream targets. An enrichment of terms associated with ABC transport, ion transport, and phosphate transport was observed. Both of these parallel bioinformatic approaches led to the same conclusion: RR_1586 appears to regulate genes involved in ion transport, particularly phosphate ion transport.

### Experimental evaluation of putative regulon.

**FIG 3:**
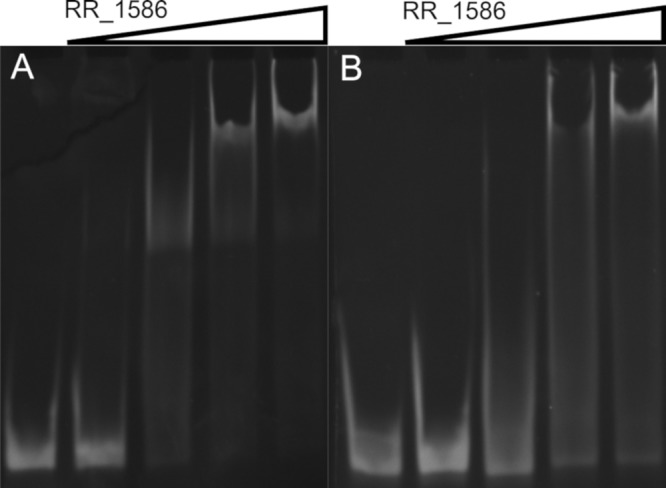
*In vitro* validation of conserved binding sites. Titrations of RR_1586 against predicted genomic binding sites upstream of *CDR20219_3145* (A) and *CDR20291_3121* (B) with 0 or 2 nucleotides, respectively, mismatching the search model were tested. Each gel shows 500 nM DNA alone and in the presence of 1×, 5×, 10×, and 20× molar equivalents of RR_1586. The oligonucleotide sequences are listed in Table S2 in the supplemental material.

The *in vitro* binding of RR_1586 to all the promoter regions of genes listed in Table 1 was confirmed using EMSAs. Figure 3 shows the titration of RR_1586 against an ideal binding site upstream of *CDR20291_3145* (Fig. 3A) and a binding site with several mismatches upstream of *CDR20291_3121* (Fig. 3B), both of which were identified to be part of the conserved RR_1586 regulon.

**FIG 4:**
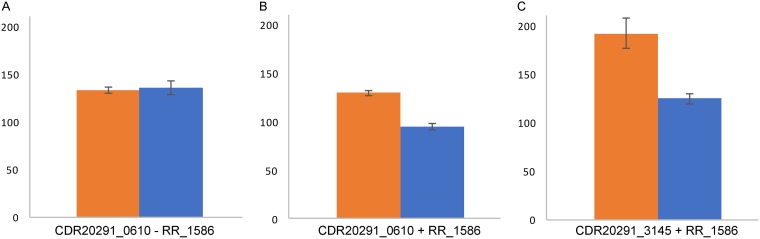
Expression of GFP from C. difficile R20291 promoters in response to RR_1586. Cell density-normalized fluorescence (relative fluorescence units/optical density at 600 nm, plotted on the *y* axis) of GFP was observed in E. coli Rosetta cells transformed with a reporter plasmid and/or an RR_1586 expression vector (indicated below each graph). Samples were recorded in the absence (orange) and presence (blue) of 40 μM IPTG, used to induce production of RR_1586. IPTG had no effect on fluorescence in the absence of the RR_1586-harboring vector (A), but a decrease in fluorescence was observed for vectors reporting transcription from the *CDR20291_0610* (B) and *CDR20291_3145* (C) promoters.

To evaluate the potential for regulatory interactions at these binding sites, several putative target promoters were tested in a GFP reporter assay in E. coli. The promoter regions from C. difficile R20291 genes, including at least 1 codon and up to 10 codons of the open reading frame, were cloned in frame with superfold GFP (21). E. coli expressed GFP from the tested promoters, while the induction of RR_1586 repressed expression of this GFP reporter gene (Fig. 4). These results support the hypothesis of transcriptional regulation by RR_1586 at these identified sites.

### Effects of phosphorylation on oligomerization and DNA binding.

**FIG 5:**
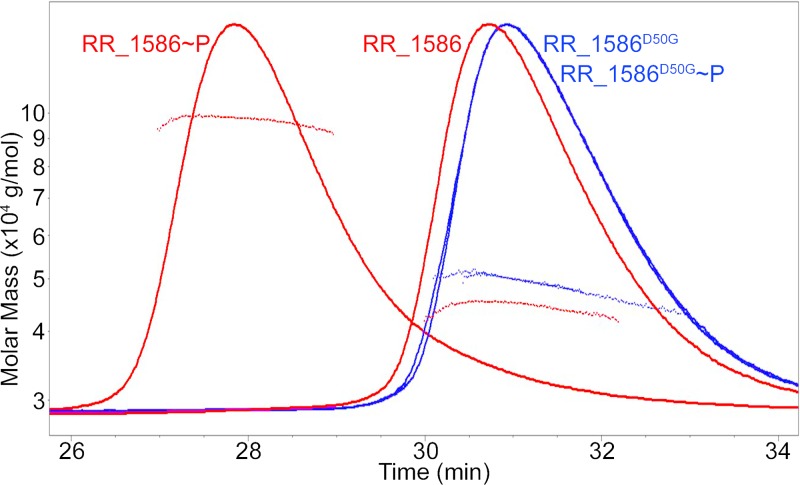
Phosphorylation dependence of oligomeric state analyzed by SEC-MALS. SEC elution (curves) and light-scattering (dots) profiles are shown. Addition of the small-molecule phosphodonor phosphoramidate shifts the molecular weight of wild-type RR_1586 (red) from 57.5 to 119 kDa. In contrast, the apparent molecular weight of nonphosphorylatable RR_1586^D50G^ (blue) shifts the molecular weight only from 59.4 to 52.6 kDa. Monomeric RR_1586 is expected to be 28 kDa.

The results presented thus far define the components of a putative RR_1586 regulon but provide little evidence for the mechanisms governing regulation. OmpR family proteins are often monomeric and form dimers upon phosphorylation to promote binding to their genomic targets (22, 23). We observed, however, that RR_1586 purifies as a dimer and shifts to an apparent tetrameric species in the presence of a small-molecule phosphoryl donor, phosphoramidate (PA) (Fig. 5), as judged by multiangle light scattering (MALS) in line with size exclusion chromatography (SEC) (SEC-MALS). RR_1586 with the phosphorylatable aspartate Asp50 mutated to a glycine (D50G) was not affected by PA, indicating that the dimer-to-tetramer shift was dependent on phosphorylation of the active-site aspartate. Secondary structure analysis by protein Fourier-transform infrared spectroscopy (24) showed no significant difference between the wild-type and D50G proteins, validating RR_1586^D50G^ as a well-folded, phosphorylation-negative control (data not shown).

**FIG 6:**
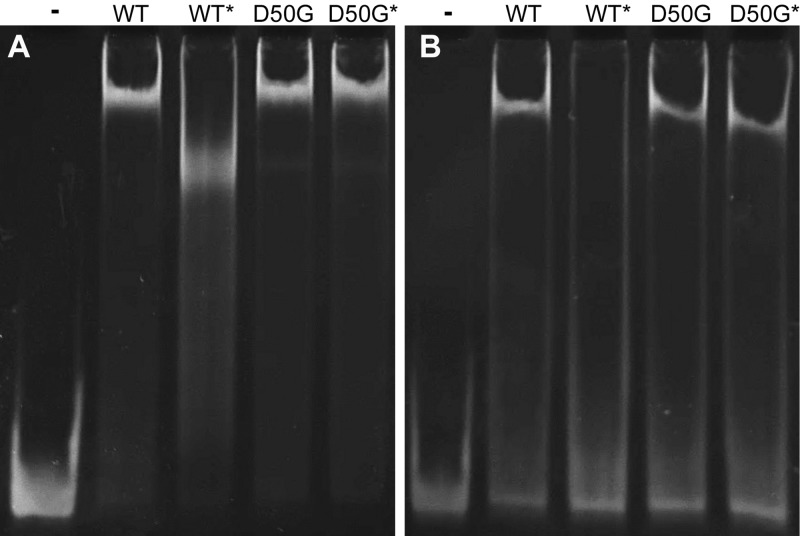
Phosphorylation dependence of DNA binding also depends on DNA sequence. The presence (*) of phosphoramidate has only minor effects on RR_1586 binding to a site upstream of *CDR20291_2142* (A) but disrupts binding to sites with a mismatch to the B1H-derived motif, such as the one upstream of *CDR20291_1583* (B). In both cases, use of the phosphorylation mutant RR_1586^D50G^ reverses these effects. The oligonucleotide sequences are listed in Table S2 in the supplemental material.

We also tested the effects of phosphorylation on DNA binding by the addition of PA to the EMSA reaction buffer. The most striking effect was that phosphorylation diminished binding to sites that do not perfectly match the RR_1586 binding site, such as the one found upstream of *CDR20291_1583* (Fig. 6B). PA could disrupt binding through ionic interaction with the RR_1586 DBD or DNA. However, all effects of PA were reversed by using RR_1586^D50G^ (Fig. 6), demonstrating the importance of phosphorylation of the active-site aspartate. Binding to ideal sites, such as the one upstream of *CDR20291_2142*, was not disrupted by phosphorylation (Fig. 6A). The amplitude of the electrophoretic shift was altered in some cases, although the convoluted effects on shape and charge make it impossible to reliably interpret the significance of such a change (25). These results show that phosphorylation-dependent changes in binding occur in a sequence-dependent manner.

## DISCUSSION

One of the goals of this work was to accelerate the study of gene regulation by two-component systems in Clostridioides difficile, particularly the hypervirulent R20291 strain. Methods in synthetic biology and bioinformatics were used as a framework to predict the direct regulon of RR_1586. RR_1586 is encoded in a five-open-reading-frame operon including *CDR20291_1583* to *CDR20291_1587*, annotated as a hypothetical protein, a putative DNA-binding protein, a putative lipoprotein, an RR, and an HK, respectively. Of these genes, *CDR20291_1586* (encoding RR_1586) is reported to be essential for sporulation, and the ortholog of *CDR20291_1583* was differentially regulated to a detectable degree during germination of the C. difficile 630 strain (15, 26). The other genes in this operon were not identified in these reports, which tested the whole genome at an apparently single-gene resolution. This potential connection to a biologically and medically important phenotype and the lack of any other relevant information made RR_1586 a suitable target for this study.

### Extension of the B1H findings to genomic context.

The hypothesis of self-regulation can be a very useful starting point for identifying downstream targets of transcription-regulating RRs (27), but initial attempts to observe the *in vitro* binding of RR_1586 to regions upstream of the *CDR20291_1583* gene and neighboring operons failed to detect binding (data not shown). The B1H assay employed here screens for binding to a large library of randomized DNA sequences in parallel (16). It depends primarily on the design of a suitable DBD fusion construct and not on the accuracy of an initial hypothesis. Several versions of a B1H assay have been applied to RRs (28–31). However, to our knowledge only two RRs have been characterized using the improved, ωRNAP fusion-based assay; both studies used full-length RRs (29, 30). B1H selection was unsuccessful using the full-length RR_1586, but one DBD construct in our series of three met the conditions for successful selection and identification of a specific motif. With the aid of an empirically derived DNA-binding specificity motif, we found that RR_1586 does indeed bind upstream of its own operon *in vitro* (Fig. 6). The confirmed binding site partially extends into the coding region, which was not included in initial tests. This anecdote exemplifies the utility of a B1H screen for precisely defining potential genomic binding sites. This approach, predicting downstream targets from an observed specificity motif, circumvents the need for genetic manipulation required for approaches where binding sites are predicted among differentially expressed genes.

The DNA motif derived in the B1H assay is not a direct representation of the genetic regulatory element recognized by RR_1586 in C. difficile. The 5 to 7 base-specific and 3 AT-rich positions and the overall length of the RR_1586 specificity motif are consistent with the binding of a monomeric RR, but OmpR family response regulator proteins often bind direct repeats (22). Furthermore, the B1H assay utilizes a synthetic library coupled to a synthetic signaling pathway, and we were able to identify a motif only under conditions that excluded low-activity binding sites (32). This is in contrast to consensus motifs derived from transcriptomics data, which represent coevolving interactions between protein and DNA elements tuned to the needs of the cell. These considerations led us to rely on comparative genomics strategies to identify putative gene targets.

Evolutionary conservation of regulatory function implies that the response regulator, its downstream target genes, and their respective binding sites will likely be conserved. We evaluated every gene in the C. difficile R20291 genome for the possibility that it fits these conditions of conservation among a set of Peptostreptococcaceae genomes. This search identified operons, including the *speADEB* and *potABCD* operons, encoding spermidine biosynthesis and transport pathways, respectively. Many ABC transporter systems, and particularly *potABCD*, have been reported to be important for sporulation and/or germination (15, 26). RR_1586 binding sites are statistically correlated (*q* < 0.05) to gene ontology terms referencing ion transport and ABC-type transporter systems, suggesting that a possible role of RR_1586 is to regulate the transport of ions. These conclusions may explain why *CDR20291_1586* was found to be essential for sporulation in a high-throughput screen (15), given that inorganic phosphate induces sporulation in Clostridium perfringens (33).

### Interpretation of phosphorylation-dependent regulation.

The main driver of two-component signal transduction is phosphoryl transfer between an HK and an RR. Phosphorylation of RR_1586 results in changes of oligomeric state and DNA binding, implying possible regulatory mechanisms. Binding to an RR_1586 binding site positioned from −17 to +4 relative to the annotated translational start site of *CDR20291_1583* likely inhibits the advancement of the transcriptional machinery and represses the expression of downstream genes, including *CDR20291_1586*. Phosphorylation of RR_1586 disrupted binding to this position *in vitro*, suggesting a potential feedback loop wherein RR_1586 represses self-expression until it becomes phosphorylated. This theme of phosphorylation-driven release of binding was repeated across most of the tested binding sites.

**FIG 7:**
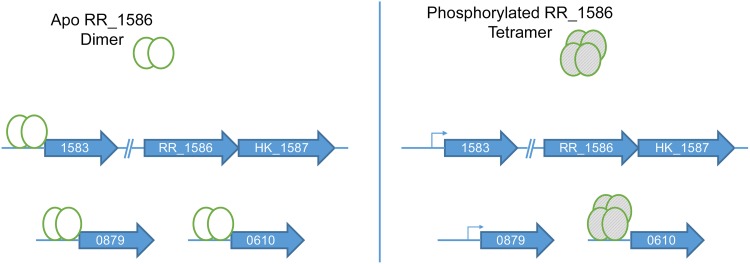
Working model of the putative RR_1586 regulon. RR_1586 binds a larger set of predicted sites as a dimeric apo protein than it does as a phosphorylated tetramer. Both forms of RR_1586 bind to ideal sites, such as one upstream of *CDR20291_0610*. Binding sites such as those upstream of *CDR20291_0879* (the leader of the operon encoding the PotABDE system) and *CDR20291_1583* (the leader of the operon encoding RR_1586 and partner HK_1587) are bound by apo RR_1586 but not by phosphorylated RR_1586. The position of the *CDR20291_1583* binding site (from positions −17 to +4 relative to the translational start site) suggests that binding would disrupt transcription, thus creating a phosphorylation-dependent transcription feedback loop.

Reporter assays showed that expression of RR_1586 repressed expression of GFP from *CDR20291_0610* and *CDR20291_3145* promoters, which encode perfect matches to the RR_1586 consensus binding sites. Although the magnitude of repression is relatively low in E. coli, we anticipate a greater effect in native C. difficile. *In vitro* binding to these sites and to the other ideal RR_1586 binding sites was not affected by phosphorylation. Regulation of these genes would be subject to changes in expression of RR_1586, which is potentially mediated by the phosphorylation-dependent self-regulation described above. Because the phosphorylation state is likely to change more rapidly than the protein concentration, our results may also point to temporal differences in the regulation of gene targets. The themes of regulatory mechanisms of the RR_1586 regulon are depicted in Fig. 7. We propose that the differential binding between dimeric apo-RR_1586 and tetrameric phospho-RR_1586 suggests both phosphorylation-dependent and -independent regulatory mechanisms for different gene promoter targets. These two mechanisms appear to correlate to binding sites with low and high identity to the B1H-derived binding site, respectively.

Phosphorylation-dependent changes in binding may reflect the conformational states accessible to apo and phosphorylated forms of the protein. One model of RR activation proposes that phosphorylation restricts the receiver domain conformation from a mixed population to a nearly homogeneous population of an activated conformational state (34). As phosphorylation stabilizes one conformation, the absence of other conformations may preclude binding to certain DNA sequences. On the other hand, rather than constricting sequence space, phosphorylation may shift the center of sequence space recognized by RR_1586. This would manifest as changes in the DNA-binding specificity motif upon phosphorylation. We are actively studying this possibility and look forward to *in vivo* verification of these conclusions.

Finally, we emphasize the reliability of this experimentally informed bioinformatics approach. For the samples that we tested, *in vitro* binding was accurately indicated if the site surpassed statistical thresholds set in the bioinformatic searches. This is not surprising, considering that the B1H assay selects for preferential binding to a 28-bp sequence in competition with the entire E. coli genome, a simulation of the selectivity required for regulation in the native host. Similarly, using bioinformatic constraints to identify conserved binding sites simulates the evolutionary conservation of function, identifying targets most likely to be functionally conserved. Both the B1H and GFP reporter assays were performed without purified protein, meaning that a putative regulon could potentially be identified and initially validated even for proteins that are not amenable to overexpression and purification. We anticipate that this study will serve as a model for analysis of two-component gene regulation in C. difficile and other pathogenic bacteria.

## MATERIALS AND METHODS

The sources of the strains and plasmids used in this work are listed by their associated method in Table 2. The custom DNA oligonucleotides and primers (Sigma) used in this study are listed in Table S2 in the supplemental material. EmeraldAmp GT master mix (Clontech) was used for all PCRs unless explicitly stated. ZymoPURE midiprep, DNA Clean & Concentrator 5 (DNA C&C), and Oligo Clean & Concentrator (Oligo C&C) kits were purchased from Zymo Research. Restriction enzymes, ligases, and RecA were purchased from NEB. Sequencing was performed by the Oklahoma Medical Research Foundation DNA Sequencing Core facility.

**TABLE 2:** Strains and plasmids used in this work

Strain or plasmid	Use	Source (Addgene no.)
pSGC-RR_1586	Protein expression	Steve Almo
pSGC-RR_1586_D50G	Protein expression	This work
USO *hisB pyrF rpoZ* mutant (E. coli)	B1H (selection)	Scot Wolfe (18049)
USO *hisB pyrF* mutant (E. coli)	B1H (counterselection)	Scot Wolfe (12614)
pH3U3-mcs	B1H (prey)	Scot Wolfe (12609)
pB1H2w2-Zif268	B1H (positive control)	Scot Wolfe (18045)
pB1H2w2-Prd	B1H (cloning template)	Scot Wolfe (18038)
pB1H2w2-mutOdd	B1H (negative control)	Scot Wolfe (18044)
pB1H2w2-1586_FL	B1H (selection)	This work
pB1H2w2-1586_R124	B1H (selection)	This work
pB1H2w2-1586_S131	B1H (selection)	This work
pB1H2w2-1586_Q151	B1H (selection)	This work
pJKR-L-tetR	GFP reporter	George Church (62562)
p0610-GFP	GFP reporter	This work
p3145-GFP	GFP reporter	This work

### Preparation of RR_1586 and derivatives.

Hexahistidine-tagged RR_1586 was purified for *in vitro* analysis using nickel affinity and size exclusion column chromatography. Proteins were expressed in BL21(DE3) Rosetta cells from pSGC plasmids constructed in the laboratory of Steve Almo at the Albert Einstein College of Medicine. Cells were lysed by sonication in 20 mM HEPES, pH 7.5, 300 mM NaCl, 20 mM imidazole, and 5% glycerol. The lysate was loaded onto a 5-ml hand-poured MCLAB Ni-nitrilotriacetic acid column and washed with lysis buffer. RR_1586 was eluted in lysis buffer with increasing imidazole concentrations in 100 mM steps. Size exclusion chromatography was performed in the OU Protein Production Core facility using a 24-ml Superdex 200 Increase column (GE Healthcare) with 20 mM HEPES, pH 7.5, and 150 mM NaCl. Protein concentrations were estimated using a Bio-Rad protein assay reagent standardized against bovine serum albumin. Phosphorylated RR_1586 was obtained by incubating 50 μM pure protein with 50 mM phosphoramidate in 20 mM Tris, pH 8, 50 mM NaCl, 10 mM MgCl_2_ for 10 min at room temperature. Phosphoramidate was synthesized following published procedures (35).

Primer-directed mutagenesis was used to replace the RR_1586 phosphorylatable aspartate with glycine (D50G). The primers used are listed in the supplemental material (Table S2). LongAmp *Taq* 2× master mix (NEB) was used as recommended by the manufacturer to incorporate the mutation during whole-plasmid PCR amplification. The mutation was confirmed by sequencing. The RR_1586^D50G^ protein was expressed and purified as described above for wild-type RR_1586.

### Multiangle light scattering and protein Fourier-transform infrared spectroscopy.

A MiniDawn Treos (Wyatt) multiangle light-scattering instrument in line with a Superdex 200 Increase SEC column was used to measure the molar masses of purified RR_1586 and its derivatives. We also measured the infrared absorbance spectra of RR_1586 and RR_1586^D50G^ to detect changes in the amide I and amide II bands associated with the protein secondary structure. We used a Bruker Confocheck Tensor II instrument fitted with an AquaSpec sample cell (Bruker), and the temperature was regulated by use of a Ministat 125 (Ruber) water bath set to 23°C. Protein was equilibrated into 20 mM HEPES, pH 7.5, and 150 mM NaCl by SEC before analysis. Opus (version 7.5) software was used to evaluate secondary structure features using the manufacturer's protocols.

### Construction of a prey plasmid library and bait plasmids for bacterial one-hybrid assay.

A library of plasmids harboring *his3* and *ura3* expressed under the control of randomized 28-mers was constructed based on published methods (36). The strand complementary to a commercial 71-mer oligonucleotide was synthesized by PCR. NotI restriction fragments were separated on a 20% polyacrylamide gel. The larger band was excised and digested with EcoRI. Final purification by a Oligo Clean & Concentrator kit retained the desired sticky-ended 28-mer library but not the 6-nucleotide by-product. The insert was ligated into pH3U3-mcs, and the resulting library was transformed directly into the counterselection strain. We performed counterselection three times using liquid gel medium instead of solid agar (37). The final plasmid library was tested using pB1H2w2-mutOdd and pB1H2w2-Zif268 control plasmids.

The omega subunit of RNAP was fused to full-length RR_1586 and three constructs of its DNA-binding domain (at positions Arg124, Ser131, and Gln151) using sequence- and ligation-independent cloning (38). PCR amplification of vector pB1H2w2-Prd (Long-Amp *Taq* 2× master mix; NEB) and the insert introduced complementary overhangs to be recombined *in vitro* by RecA. Plasmid construction was confirmed by Sanger sequencing. Plasmid DNA from overnight cultures in 50 ml of LB was isolated and concentrated by ethanol precipitation in preparation for selection (36).

### Bacterial one-hybrid selection and data analysis.

Selection proceeded as described previously (16). The RR_1586 bait and prey libraries were cotransformed by electroporation into USO cells and plated onto minimal medium lacking histidine and uracil at a density of between 5 × 10^4^ and 5 × 10^5^ CFU/cm^2^. The plates were wrapped with Parafilm M and incubated at 37°C until the colonies were picked for colony PCR. The PCR product was purified by phenol-chloroform extraction and sequenced. The MEME suite (v4.12.0) was used to identify overrepresented motifs in RR_1586-selected sequences (39). Zero or one instance of the motif was allowed per sequence (−zoops) on the given or complementary strand (−revcomp) with a minimum motif width of 3 nucleotides. An E value threshold of 0.005 was set. All other parameters were left at the default.

### Genome scanning and comparative genomics.

The accession numbers for the genome assemblies analyzed in this study are listed in Table S3. The pattern-search and footprint-scan scripts from the Regulator Sequence Analysis Tools suite (RSAT) were used for single-genome scans and comparative genomics approaches (17). Search models for the direct repeat were constructed by duplicating the first 11 positions in the search strings or matrices.

Multispecies GOMo, part of the MEME suite, was used to identify statistically significant correlations between promoters with RR_1586 binding sites and gene ontology (GO) terms associated with downstream genes from 13 Peptostreptococcaceae genomes with RRs highly similar to RR_1586 (20). The GO terms assigned to C. difficile R20291 proteins by BLAST2GO were also applied to their respective orthologues (40). A union of terms from members of an operon was assigned to the leading gene to account for the species-specific operon structure. Orthology and operon structure were inferred using RSAT (17), except that C. difficile R20291 operon predictions are from the DOOR2 database (19). GO maps for all genomes were combined into a single input file for GOMo analysis (20).

### Electrophoretic mobility shift assays.

Binding of full-length RR_1586 to DNA was observed *in vitro* using EMSAs. Pairs of synthetic single-stranded oligonucleotides (Table S2) in 10 mM Tris, pH 8, 50 mM NaCl were annealed at 95°C for 5 min and passively cooled to room temperature. Titrations of protein and 5 pmol DNA in 10 μl of 10 mM Tris, pH 8, 50 mM NaCl, and 10 mM MgCl_2_ were incubated at room temperature for 10 min, and then 5 μl of 50% glycerol was added to aid in loading. Samples were loaded onto prerun 10% polyacrylamide gels with 0.5× Tris-borate-EDTA (TBE) as the running buffer. Gels were run at 120 V for 1 h with the gel box submerged in ice. DNA was stained by rocking the gel in running buffer spiked with ethidium bromide for 5 min. Images were captured using a Gel Logic 100 system with a UV transilluminator.

### Recombinant reporter assay.

The tetracycline biosensor plasmid pJKR-L-tetR (21) was repurposed as a GFP reporter of transcription in E. coli. Restriction sites were introduced to replace the existing ribosomal binding site with inserts spanning the upstream region and the first few codons of C. difficile R20291 genes. TetR promoters were kept intact to serve as an anhydrotetracycline-inducible control of GFP expression and to screen for properly integrated inserts. All vectors were confirmed by sequencing.

RR_1586-dependent expression of GFP was tested in E. coli BL21(DE3) Rosetta cells. Saturated overnight cultures were diluted 100-fold in fresh LB and shaken at 37°C for 3 h. RR_1586 expression was then induced with 40 μM IPTG (isopropyl-β-d-thiogalactopyranoside). Cell growth and GFP fluorescence were monitored as described previously (21). Cell density-normalized fluorescence at the final 15-h time point is reported.

## Supplementary Material

Supplemental file 1
