# Supplementary material for: Regulatory Targets of the Response Regulator RR_1586 from Clostridioides difficile Identified Using a Bacterial One-Hybrid Screen
Source: J Bacteriol. 2018 Nov 6;200(23):e00351-18. doi: 10.1128/JB.00351-18 (PMC6222199; doi:10.1128/JB.00351-18)
Supplement: Supplemental file 1 [file zjb999094915s1.pdf]

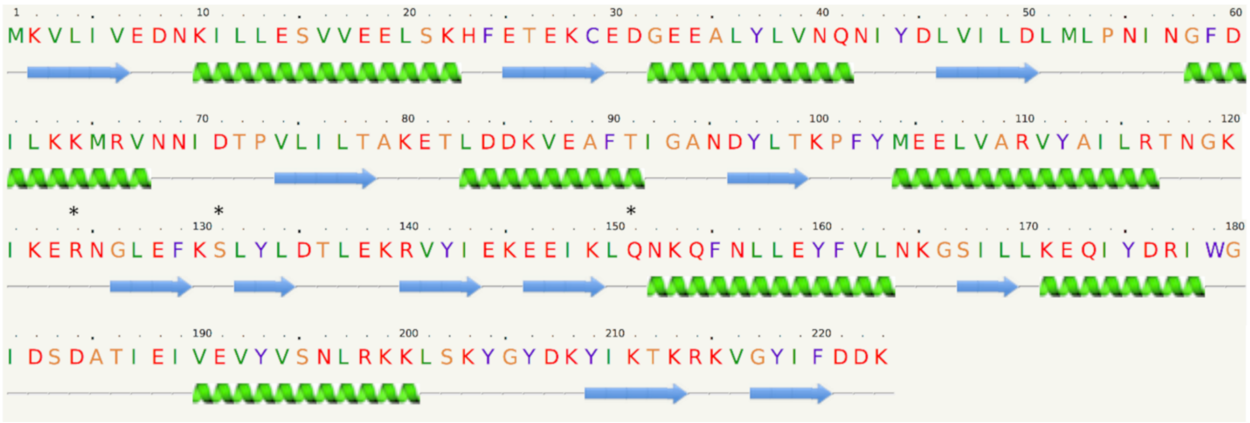

**Figure S1** Secondary structure of RR_1586 as predicted by the Phyre2 server (1). The amino acid residues where the RNA polymerase ω subunit was fused to the RR_1586 DNA-binding domain are indicated (*) at Arg124, Ser131, and Gln151. The Ser131 construct includes the winged helix-turn-helix domain and most of the β platform, excluding the flexible portion of the inter-domain linker. The Arg124 construct adds the flexible linker, and the Gln151 excludes the β platform.


1. Kelley LA, Mezulis S, Yates CM, Wass MN, Sternberg MJ. 2015. The Phyre2 web portal for protein modeling, prediction and analysis. Nat Protoc 10:845-58. https://doi.org/10.1038/nprot.2015.053

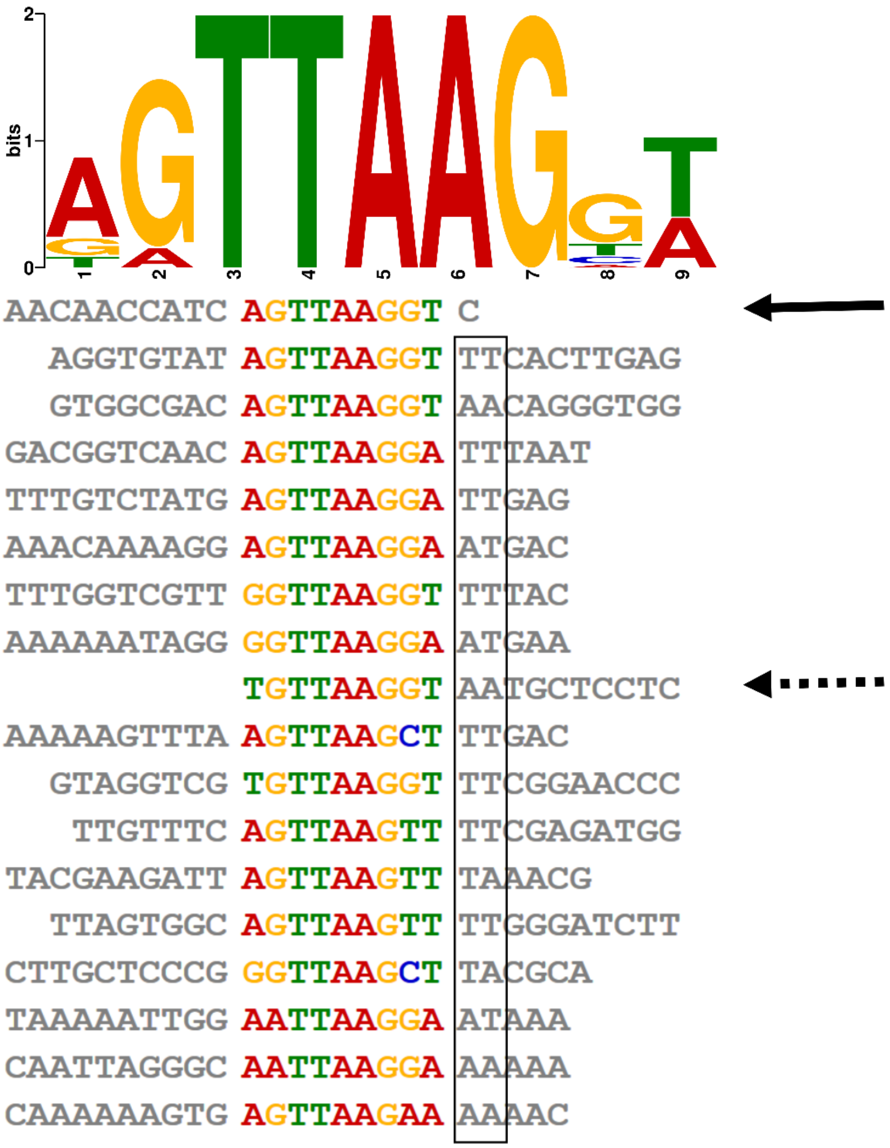


**Fig S2.** Data analysis for motif discovery.

A nine nucleotide motif was identified by MEME in the raw selected sequences. The conserved nucleotides contributing to the motif are colored, but two more nucleotide positions (boxed) have conserved A or T nucleotides. These nucleotides were excluded from the motif because of a penalty applied for reaching the end of the sequence indicated by a solid arrow. It is possible that RR_1586 bound a region straddling the border between the vector and randomized insert. We therefore included six nucleotides of the adjacent vector sequence in our final analysis to achieve the logo presented in Figure 1. Full sequences are shown in Table S1. Adding vector nucleotides to the sequence marked with a dashed arrow did not significantly change the conserved motif and was therefore left unchanged.

**Table S1** Random inserts isolated under high stringency or low stringency selection conditions.[a]

| Insert Sequence | Stringency |
|---|---|
| GAGGACAACAGCTTTGGGTACTTGAAAA | High |
| TACCTTGCTCCCGGGTTAAGCTTACGCA | High |
| GAAGATCCCAAAACTTAACTGCCACTAA | High |
| GCCACCCTGTTACCTTAACTGTCGCCAC | High |
| ACTCAAGTGAAACCTTAACTATACACCT | High |
| GTATTTTTGTGCGTTTGATTTTTTTTTG | High |
| **cggccgc**GACCTTAACTGATGGTTGTTAAAACACG[b] | High |
| TGGGTTCCGAAACCTTAACACGACCTAC | High |
| TTTTTTTGTGTGTTCTTGTTTTTTTTTT | High |
| TAAAAAAAAGTTTAAGTTAAGCTTTGAC | High |
| ATCTACGAAGATTAGTTAAGTTTAAACG | High |
| TAAAAAACAAAAGGAGTTAAGGAATGAC | High |
| TTTTTGTCTATGAGTTAAGGATTGAG | High |
| TCTGTTTGGTCGTTGGTTAAGGTTTTAC | Low |
| CCTACAAAAAAGTGAGTTAAGAAAAAAC | Low |
| AGGGTGGGTGTGGGTGTATTGTGGGTGG | Low |
| TTTGACGGTCAACAGTTAAGGATTTAAT | Low |
| AAAAAAATACAAGTAGACAAGAATGACG | Low |
| TGTTAAGGTAATGCTCCTCACTTGTGCC | Low |
| TCGGTCATTGTTGGGGTTGGGGGGGGGG | Low |
| CTCCTGGATGGCTGTTTGACTTTGCGTT | Low |
| TACACAATTAGGGCAATTAAGGAAAAAA | Low |
| GAGCTTTTTGGGGGGTCGGACTGTTTGC | Low |
| TATATAAAAATTGGAATTAAGGAATAAA | Low |
| AACCAGACGCCCGCGAACGACAAATAAA | Low |
| AATCAAAAAATAGGGGTTAAGGAATGAA | Low |
| TGAAACGATCGTCAATTGACTTCACGCC | Low |
| ATATGAGAGGAGAAGGGCAGAAATCAAT | Low |
| TAGTGTATTTGTTGATGTTTGTTTG | Low |
| AGCCATCTCGAAAACTTAACTGAAACAA | Low |

[a] High stringency is defined as 20 mM 3AT, and low stringency is defined as 10 mM 3AT in the selection medium.

[b] Bolded text indicates sequence outside the randomized regions. See Figure S2 for more details.

**Table S2** Oligonucleotides reported in this study.

| ID | Sequence[a] | Experiment[b] |
|---|---|---|
| **pr117_F** | GTTATTTTAGMGTTAATGCTTCCTAATATAAATG | RR_1586 D50G |
| **pr118_R** | AGCATTAACMCTAAAATAACTAAATCATAT | RR_1586 D50G |
| **pr21_F** | GGCGCGAATTCGNNNNNNNNNNNNNNNNNNNNNNNNNNNGCGGCCGCAAGGTAGCTGATTCCGTTCTCGC | B1H library synthesis |
| **pr22_R** | GCGAGAACGGAATCAGCTACCTT | B1H library synthesis |
| **pr23_R** | GCCGCTGTTGGTACCATG | pB1H2w2 amplification |
| **pr24_F** | AAGCTTGGAGCCACCCGCGGAGCAGCTGCCTCAGTG | pB1H2w2 amplification |
| **pr25_R** | GCGGGTGGCTCCAAGCTTCCGTATCCACCTTTACTG | B1H: RR_1586 insert |
| **pr122_F** | CATGGTACCAACAGCGGCTCTTTATATTTAGATACACTAG | B1H: RR_1586 DBD (Ser131) insert |
| **pr123_F** | CATGGTACCAACAGCGGCCAAAACAAACAGTTTAACTTG | B1H: RR_1586 DBD (Gln151) insert |
| **pr28_F** | CATGGTACCAACAGCGGCAGAAACGGATTAGAATTTAAAT | B1H: RR_1586 DBD (Arg124) insert |
| **pr33_F** | GGACTACAAGGATGACGACGAC | B1H ω fusion sequencing |
| **pr31_F** | CAAATATGTATCCGCTCATGAC | B1H colony PCR and sequencing primer |
| **pr176_R** | CCAGAGCATGTATCATATGGTCCAGAAACCC | B1H colony PCR |
| **pr156_F** | ATTTTACACGAAATGGGCACGAAGTATAC | *CDR20291_0578*, double subsituted |
| **pr157_R** | GTATACTTCGTGCCCATTTCGTGTAAAAT | *CDR20291_0578*, double subsituted |
| **pr154_F** | ATTTTATTAAGAATGGGTTAAGAGTATAC | *CDR20291_0578*, native |
| **pr155_R** | GTATACTCTTAACCCATTCTTAATAAAAT | *CDR20291_0578*, native |
| **pr158_F** | ATTTTATTAAGAATGGGCACGAAGTATAC | *CDR20291_0578*, single substituted |
| **pr159_R** | GTATACTTCGTGCCCATTCTTAATAAAAT | *CDR20291_0578*, single substituted |
| **pr191_F** | TTGCTTGTTTAAGACATACTTAAATTTTAGG | *CDR20291_1833* inverted repeat |
| **pr192_R** | CCTAAAATTTAAGTATGTCTTAAACAAGCAA | *CDR20291_1833* inverted repeat |
| **pr188_F** | ATAGTTAAGGTTTAATTAAGATTAAA | *CDR20291_3145* |
| **pr189_R** | TTTAATCTTAATTAAACCTTAACTAT | *CDR20291_3145* |
| **pr268_F** | CTATATTAGGATTAAGTTAAGCAAGTGT | *CDR20291_3121* |
| **pr269_R** | ACACTTGCTTAACTTAATCCTAATATAG | *CDR20291_3121* |
| **pr225_F** | AGGAATTAAGGAGCAATTAAATGATG | *CDR20291_1583* |
| **pr226_R** | CATCATTTAATTGCTCCTTAATTCCT | *CDR20291_1583* |
| **pr291_F** | TCGGTACCGAAAAGGAAGAGCTAGAAAAAAGAC | *CDR20291_3145* upstream for GFP reporter |
| **pr292_R** | AGTAAGCTTCATCTCATACCACTCCCTATC | *CDR20291_3145* upstream for GFP |

| | | |
|---|---|---|
| | | reporter |
| **pr239_F** | CATGGTACCAACAGCGGCATCATTTTCATTTCAACTAAATTTG | *CDR20291_0610* upstream for GFP reporter |
| **pr240_R** | GCGGGTGGCTCCAAGCTTCATATTCACACCTCAGG | *CDR20291_0610* upstream for GFP reporter |
| **pr241_F** | TCGGTACCAGTAAGCTTATGCGTAAAGGTGAAGAACTG | Adapt pJKR-L-tetR for GFP reporter |
| **pr242_R** | ACTGGTACCGAATTCGGTCATGCGTCC | Adapt pJKR-L-tetR for GFP reporter |
| **pr243_F** | GACGGCACGTACAAAACCCGTG | Adapt pJKR-L-tetR for GFP reporter |
| **pr244_R** | TGTACGTGCCGTCGTCTTTGAAAGAG | Adapt pJKR-L-tetR for GFP reporter |
| **pr245_F** | GAGATACTGAGCACATCAGCAGG | GFP reporter sequencing primer |

[a] The conserved TTAAG, or substitute nucleotides, are underlined for oligoes used in Figure 2.

[b] CDR20291 locus tags indicate nearest downstream gene from RR_1586 binding site.

**Table S3** Accession numbers of genome assemblies used in RSAT footprint-scan.

| Organism | RefSeq Assembly | Orthologue[a] |
|---|---|---|
| *Acetoanaerobium noterae* | GCF_900168025.1 | - |
| *Acetoanaerobium sticklandii* | GCF_000196455.1 | - |
| *Asaccharospora irregularis* | GCF_900129815.1 | + |
| *Clostridioides difficile QCD-66c26* | GCF_000003215.1 | + |
| *Clostridioides mangenotii* | GCF_000498755.1 | + |
| *Clostridioides difficile R20291* | GCF_000027105.1 | + |
| *Criibacterium bergeronii* | GCF_001693775.1 | + |
| *Filifactor alocis* | GCF_000163895.2 | + |
| *Intestinibacter bartlettii* | GCF_000154445.1 | + |
| *Paeniclostridium sordellii* | GCF_000444095.1 | + |
| *Paraclostridium benzoelyticum* | GCF_001006285.1 | + |
| *Paraclostridium bifermentans* | GCF_000452225.2 | + |
| *Peptoanaerobacter stomatis* | GCF_000238095.2 | - |
| *Peptoclostridium acidaminophilum* | GCF_000597865.1 | - |
| *Peptoclostridium litorale* | GCF_000699585.1 | - |
| *Peptostreptococcaceae bacterium AS15* | GCF_000287695.1 | - |
| *Peptostreptococcaceae bacterium VA2* | GCF_000686145.1 | + |
| *Peptostreptococcaceae bacterium oral taxon 113* | GCF_000467935.1 | + |
| *Peptostreptococcus anaerobius* | GCF_000178095.1 | + |
| *Peptostreptococcus stomatis* | GCF_000147675.1 | + |
| *Proteocatella sphenisci* | GCF_000423525.1 | - |
| *Romboutsia timonensis* | GCF_900106845.1 | + |
| *Tepidibacter formicigenes* | GCF_900142235.1 | - |
| *Tepidibacter thalassicus* | GCF_900129915.1 | - |
| *Terrisporobacter glycolicus* | GCF_000373865.1 | + |
| *Terrisporobacter othiniensis* | GCF_000808015.1 | + |

[a] Indicates if an orthologue of RR_1586 was (+) or was not (-) found in the genome.
